# Moving between ideologies in self‐management support—A qualitative study

**DOI:** 10.1111/hex.12833

**Published:** 2018-10-05

**Authors:** Dagmara Bossy, Ingrid Ruud Knutsen, Anne Rogers, Christina Foss

**Affiliations:** ^1^ Norwegian National Advisory Unit on Learning and Mastery in Health Universitetssykehus HF Aker Sykehus Oslo Norway; ^2^ Department of Nursing Science Faculty of Medicine University of Oslo Oslo Norway; ^3^ Department of Nursing and Health Promotion Faculty of Health Sciences Oslo Metropolitan University Kjeller Norway; ^4^ NIHR CLAHRC Wessex Faculty of Health Sciences University of Southampton Hampshire UK

**Keywords:** health policy implementation, health professionals, health promotion, institutional logic, self‐management support, type 2 diabetes

## Abstract

**Background:**

Reforms in current health policy explicitly endorse health promotion through group‐based self‐management support for people with long‐term conditions. Health promotion and traditional medicine are based on different logics. Accordingly, health professionals in health‐promoting settings demand the adoption of new practices and ways of thinking.

**Objectives:**

The objective of our study was to investigate how health professionals perceive the health‐promoting group‐based self‐management support that is politically initiated for people with long‐term conditions.

**Design:**

This study had a qualitative research design that included focus group interviews and was guided by a social constructivist paradigm in which group‐based self‐management was viewed as a social construction. Different logics at play were analysed through the theoretical lens of institutional logic. Discussions among participants show frames of references seen as logics.

**Setting and participants:**

We recruited health professionals from group‐based health‐promoting measures for people with type 2 diabetes in Norway. Two focus groups comprising four and six participants each were invited to discuss the practices and value of health promotion through group‐based self‐management support.

**Results:**

The analysis resulted in three themes of discussion among participants that contained reflections of logics in movement. Health professionals’ discussions moved between different logics based on the importance of expert‐based knowledge on compliance and on individual lifestyle choices.

**Discussion and conclusion:**

The study indicates that health promotion through self‐management support is still a field “in the making” and that professionals strive to establish new logics and practices that are not considered difficult to manage or do not contain incompatible understandings.

## 1 INTRODUCTION

This study focuses on the experiences of health professionals working with self‐management support for people with type 2 diabetes and how they understand health‐promoting practices. The objective of the study is primarily health professionals’ perceptions of group‐based self‐management support. Self‐management support represents an example of a recently initiated, widely endorsed health‐promoting measure in health care.^1^, ^2^ Group‐based self‐management support is a developing field in which health professionals in interprofessional settings create and negotiate new practices.^3^ The growing prevalence of long‐term conditions has been described in the literature as a burden on public health care, represented by its costs to society and its impact on the quality of life of individuals diagnosed with one or more long‐term conditions.^4^ Hence, changes in contemporary health policy are directed at improving self‐management and reducing the costs of services,^5^ which involve a reorganization of chronic disease care delivery taking place in several European countries.^6^, ^7^, ^8^, ^9^ The new health policy affects services to people with long‐term conditions, and type 2 diabetes is a condition that affects particularly vulnerable groups in society.^10^, ^11^, ^12^ Health professionals are expected to find new ways of meeting patients within the framework of groups to support self‐management, which is different from traditional one‐on‐one consultations.

Research shows that health professionals influence the uptake and contagion of self‐management strategies for people with type 2 diabetes.^13^, ^14^, ^15^ However, some studies show that in group‐based self‐management support settings, health professionals tend to dichotomize participants into “good” or “bad” patients, such that those who do not achieve any behaviour change are viewed as difficult and noncompliant.^16^, ^17^, ^18^ Investigating what “meets” patients when they enrol in group‐based self‐management support is important. Awareness of how health professionals operate in these settings is important for conclusions that may form the basis for quality improvement and the design of measures well suited to meet the preferences and needs of different groups of patients attending group‐based self‐management support.

Part of policy development involves decentralizing health‐promoting services from specialist care to primary care.^2^, ^19^ It has previously been shown that it is difficult for primary care practitioners to focus on health promotion.^20^ One study suggests that health professionals strive to integrate the ideals of promotion, such as well‐being, but instead tend to focus on adherence to and compliance with medical advice.^20^, ^21^ By focusing strictly on risk and disease prevention, health professionals often fail to broaden their aim towards more holistically inclusive and general health promotion.^21^, ^22^ A relevant question is whether and how the alteration of frames changes the rationale of actors in the health‐promoting setting, which is why we are interested in how health professionals perceive group‐based self‐management support. Additionally, further investigation is sought regarding the notion of coproduction in the context of group‐based self‐management support between both participants and health professionals,^16^ which requires knowledge of the pervasive logics that drive practices in self‐management support settings. Investigating both patients’ and health professionals’ perspectives simultaneously is a demanding exercise, and for the sake of being able to investigate one group at a time, our group of interest in this study is health professionals.

### 1.1 The medical perspective vs the perspective of health promotion through group‐based self‐management support

To grasp the perceptions of health professionals, it seems reasonable to highlight some overall differences between traditional perspectives of medicine and a health‐promoting approach. The way we understand the medical perspective in our study presupposes a form of practice that aligns with expectations that patients follow and comply with medical directions in a manner that heeds professional power and legitimacy.^23^, ^24^ The modus operandi of the medical perspective emphasizes treatment guided by an expert and depicts the individual as someone who “must be helped” and health professionals as legitimate experts in ensuring that this takes place.^3^

In contrast to the medical perspective, health promotion may be said to encapsulate both person‐oriented and group‐oriented dimensions,^25^ which makes it reasonable to say that health promotion sees the individual as part of his/her social context. The World Health Organization (WHO) states that health promotion is aimed at empowering individuals to take control of their health, which is depicted as a process of enabling people to take increased responsibility for their own health and well‐being.^26^, ^27^ In line with this goal, health promotion is described as aiming to direct health professionals to help people through addressing the nonmedical factors of their health.^28^ Nonmedical factors in our study reflect strategies that do not follow the medical perspective (for instance, providing support beyond medical guidelines and treatment), preferably acknowledging patients’ knowledge of their own health. Group‐based self‐management support is based on an ideology of empowering participants to become active agents in their health.^29^ The group‐based approaches vary in content and may include a mix of the following group pedagogic measures: expert‐patient tutoring; discussions between leaders and participants; educational components; and content such as nutrition advice and physical activities, the latter of which could be described as a lifestyle‐oriented approach.^3^ The Norwegian structure of group‐based support ranges from public, professionally led groups to private nonprofit layperson‐driven groups, and local physical activity and nutrition programmes may be both professionally and layperson‐driven.^30^ In other countries, self‐management support is primarily based on initiatives from volunteer and patient organizations.^31^

Health professionals must adopt changes in their ways of thinking and frames of reference when meeting patients with type 2 diabetes in groups rather than in one‐on‐one consultations and in focusing on patients’ well‐being rather than their disease. With regard to health professionals’ understanding of health promotion, research has shown that health promotion is akin to health education.^32^, ^33^ As health promotion and health education often become intermingled, it seems apt to clarify the distinction between a medical‐centred approach to health education and a health‐promoting approach. In Table 1, we have highlighted theoretical differences based on the distinction between patient‐centred and medico‐centred views on health education.^34^

**Table 1:** Two approaches to patient education

	Medical perspective	Health‐promoting perspective
Health education	Health education is a means to instigate controlled behaviour, encouraging health gains by persuasion	Health education focuses on actively inviting patients to dialogue with health professionals
Eminence of knowledge	The health professional is viewed as the legitimate holder of valued, medical information that is conveyed to the patient, who absorbs the information uncritically	The boundaries between professional‐as‐teacher and patient‐as‐learner are blurred. The patient's lay health beliefs are considered of equal value as the professional's knowledge

In line with the information presented in Table 1, studies have shown that the medical perspective has been identified as a paternalistic and individualistic “behaviour‐changing approach” when applied in health‐promoting contexts.^35^, ^36^, ^37^ The main focus in patient‐provider communication seems to be disease and treatment, despite the advocacy for a health‐promoting focus.^38^

Our study conceptualizes ideologies associated with health promotion as expressions of logics, and in this study, we use the theoretical framework of institutional logic.

### 1.2 Institutional logic

At the core of the theory of institutional logic is the premise that practices and perceptions are socially constructed. Our study thus adopts a social constructivist stance, viewing logics as practices created through constantly ongoing interactions. Institutional logics refer to a set of cultural beliefs, rules and practices that shape the thoughts and behaviours of actors in settings where individuals regularly interact.^39^ Actors may challenge or maintain and produce and reproduce logics through patterns of practices and assumptions.^40^ The logics signify certain frames of reference that guide actors’ understandings and, hence, intentions with regard to the kind of practices being developed. Practices involved in chronic disease management settings may illustrate different sets of logics that may be coinciding or incompatible. Knowledge on how different practices in the health‐promoting field are incompatible or compatible is essential when countries are undergoing a change in orientation towards health promotion.^20^, ^41^ Our aim is to investigate how health professionals perceive group‐based self‐management support and what logics they are drawing upon, within the context of focus group interviews.

## 2 DESIGN AND METHODS

As the institutional logic perspective is characterized by socially constructed understandings through shared knowledge, we needed a methodological approach that enabled us to observe the construction and upholding of shared common understandings among health professionals. The focus group interview method is seen as a useful way to elicit the coconstruction of meaning in action,^42^ in which both interaction and content are available for analysis. Listening to and observing how a focus group interview evolves in context make it possible to elicit different understandings and assumptions being described by the group.^43^ We understand interaction as the underlying context of the statements expressed.^44^, ^45^

### 2.1 Recruitment

The authors established contact with the leaders of several group‐based self‐management support measures who contacted their colleagues for group interviews. The recruitment lasted for several rounds, as health professionals represent a busy informant group. Many invitations to join the group interview were refused due to heavy workloads. Two of the participants withdrew from the interview only minutes before it started, due to other assignments in their work schedule. The focus group data were gathered and analysed in 2016.

### 2.2 Participants

Our data were derived from two focus groups that included a total of 10 health professionals (additional information on group participants is given in Table 2).

**Table 2:** Group participants

Professional composition	Sex	Experience
Group 1		
1. Nutritionist	F	Participating in learning and mastery courses for type 2 diabetes patients
2. Physiotherapist	F	Works primarily with type 2 diabetes patients but has also participated in planning and conducting courses for other patient groups for those who are struggling with lifestyle‐related challenges
3. Occupational therapist	F	Works as a supervisor for group‐based measures for people with diabetes and people struggling with morbid obesity
4. Specialist diabetes nurse	F	Has been working with type 2 diabetes and is now involved with patient education for type 2 diabetes patients
Group 2		
5. Nutritionist	F	Community care programme for diabetes patients with minority backgrounds
6. Physiotherapist	F	Healthy life central to community care and involved in a local physiotherapy centre; particular experience with female type 2 diabetes patients with immigrant backgrounds
7. Specialist diabetes nurse	F	Community care health centre for immigrants with diabetes
8. General practitioner	M	Community offer for two groups of patients: musculoskeletal pain and morbid obesity; (rehabilitation programme) offers physical activity and educational courses
9. General practitioner	F	Responsible for the collaboration between specialized and community care; education for immigrants with diabetes
10. Psychiatric nurse	M	Manager at the community health and care unit; particularly oriented towards low‐threshold health‐promoting activities, such as exercise groups; works on a health‐promoting measure called “activity during daytime”

The health professionals in our study have diverse experiences with group‐based self‐management support, most of them working with type 2 diabetes. Hence, the strength of our data set is that it represents a wide range of professions that comprise relevant occupations in group‐based support measures, such as GPs, physiotherapists, specialized diabetes nurses and nutritionists.

The literature on focus groups suggests that more information may be obtained by conducting two focus groups of four participants instead of one group of eight participants.^46^, ^47^ When the interaction and discussion are the objects of study interest, the sample size is subordinate; however, the relational context and the context of statements^48^ are important. The two focus groups differed with respect to their relational context. Group 1 consisted of four participants who knew each other (most of the participants worked together), while group 2 consisted of a larger group (six participants), several of whom did not know each other at all. We recognized many of the same discussion themes in the two group interviews, and it is reasonable to believe that our questions would have triggered the same discussions in additional interviews with health professionals.

### 2.3 Group interviews

**Figure 1:**
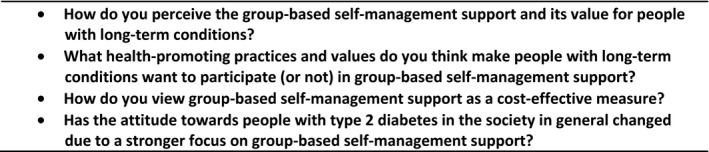
Interview guide

Two moderators led the interviews, which were conducted in Norwegian. We invited the participants to discuss the practice and value of group‐based self‐management support for people with long‐term conditions and to share their thoughts on health‐promoting policy, focusing on people with type 2 diabetes. For an overview of the research questions relevant to our analysis, see Figure 1.

At the beginning of each interview, we obtained written consent from all of the participants. The ethical approval for the research project was granted by Medical and Health Research Ethics (REK) (Grant number 2012/593).

### 2.4 Data analysis

Both focus group interviews were recorded and transcribed verbatim. We searched for the logics that comprised the assumptions, practices and values that participants expressed. In accordance with seeing logics as constructed through interaction, we also looked for agreements and disagreements. We found themes of discussion among participants in which we claim to identify the logics at play. The objective of our focus was to search for what the participants said, how they said it and how these different ways of saying it contributed to discussions.^43^, ^49^ The analysis process did not explicitly deal with subthemes, as we interpreted^50^ statements in the context of discussions to identify logics that make up the frame of reference behind the statements expressed. The main statements illustrating patterns of shared knowledge and disagreement were the basis of the overall themes.

We circulated the analysis drafts among all the coauthors and discussed them at seminars with fellow colleagues. During the analysis process, all knowledge shared in the focus group setting was seen as socially constructed^51^ and as expressions of shared logics.

## 3 FINDINGS AND INTERPRETATIONS

Both of the focus groups in our study provided rich data concerning health promotion and group‐based self‐management support. In the following sections, we start with describing the shift of frames upon which all health professionals agree, which we call “logics in movement.” Furthermore, we present how health professionals express the logic of the medical perspective through statements revealing the dominance of expert knowledge and the focus on individual responsibility. The driving force of negotiation in discussions among health professionals is seen to be lifestyle orientation.

We use numbers to categorize the individual participants. In the presentation of the findings, we connect the statements to the number that represents the participant.

### 3.1 Logics in movement

Health professionals in our focus groups acknowledged quite early in the interviews that the health‐promoting arena represents *a new setting* operating outside the “traditional” medical frame. This distinction is illuminated through the emphasis on the lack of hospital uniforms, which distinguishes a medical context from other contexts and enables the freedom to act and think differently, which is associated with an open setting and civil society. An example of such statement is the following:

It is a bit like that, I am dressed as a civilian, right. Perhaps that has something to do with it; it's not that hospital‐like. We also think it is very good that they [people with diabetes] experience it as something different as well. (4)

When seen in the context of the whole interview, the statement represents ambivalence about health professionals preferring not to “look like” health professionals and thus assuming equality with their patients. Nevertheless, group participants in our focus groups still view their duty as health professionals as giving medical advice to people with type 2 diabetes. The lack of hospital uniforms and the meetings with patients with long‐term conditions in groups embedded in a set of personal relationships instead of in one‐on‐one dyadic consultations are examples of changing frames extending beyond clinical treatment and management. The attempt to create new practices is illustrated here by civilian clothes and a “nonhospital” setting. A broader perspective encompassing the ethos of health promotion is evident in the discussion, as indicated by this quote from one of the health professionals:

As health professionals, we are so limited by thinking in terms of diagnosis! There will always be other things individuals have in common, and it helps to just to have a place to come where you can meet other people for the person you are and not the diagnosis you carry…a place that is about focusing on what opportunities and resources you have and that makes people go on with their lives and not focus on the disease! (6)

The above statement represents a high degree of self‐reflection on the medical system's focus on diagnoses as an organizing principle around which long‐term condition management revolves. The phrase “focusing on what opportunities and resources you have” may be associated with the well‐being aspect of a health‐promoting perspective. Meeting others underlines the importance of togetherness in health promotion and denotes a logic of fellowship, as has been previously pointed out in research on the self‐management of long‐term conditions and changes in health policy.^52^ As such, the statement illustrates an additional expression of logics in movement. However, even when participants reflect critically on the usage of diagnoses, they still remain attached to the medical perspective through a focus on diseases, as illustrated in the following statement:

I agree that we should focus on the activity and not the diagnosis. Different diseases may benefit from the same activities. I think it is important. And we should not talk about the disease. Instead, we should focus on creating an arena with a good social network where people can do things together that are independent of their diagnoses. (10)

The statement could be seen as representing a nascent understanding of the health‐promoting logic and new sets of practices. By distancing themselves from the concept of diagnoses, we understand that health professionals are taking a critical standpoint towards an undiluted medical approach. The statement may also be understood as rooted in the sphere of lifestyle‐oriented logic‐prioritizing lifestyle change, as it includes the words “different diseases may benefit from the same activities.” It seems here that the lifestyle dimension has become more important than the diagnosis. The distinction between diagnosis and lifestyle may be illustrative of the ambivalence underlying rootedness in a medical perspective and the simultaneous adaptation to a health‐promoting ideology. The statement is repeated, and the function of “disease” becomes clear, as shown in the following statement:

I think we should not create groups based on diagnoses. (…) I think we should focus on the activity, and then we can *put the disease under the activity*, but it is important not to talk about the disease too much. (10)

The phrase “putting the disease under the activity” illustrates the strength of the medical perspective, even though the participant is trying to search for new ways of approaching patients.

### 3.2 Priority of expert‐based knowledge

Conveying professional advice and guidelines is associated with the overall value of expert‐based knowledge. Several statements support the value of health professionals as experts, both in the practices health professionals describe and in discussions related to quality assessment. The following statement suggests that the practices used in the health‐promoting group‐based context may represent a continuation of an established medical practice centred on health education.

It is as if we have pushed the information down the patients’ throats. (Several of the group members confirm by nodding.) Perhaps they just want to spit the information back up and have nothing more to do with us. It all depends on the kind of conversation we have had with the patient. (3)

The statement is a critical reflection on the practice of health education; here, the act of conveying guidelines is reduced to “pushing the information down the patients’ throats.” Furthermore, if we view this reflection as critical of health education, it becomes clear that health professionals understand health promotion as health education that may not be effectively disseminated using existing methods. The context of the statement is relevant, as the quotation is drawn from a discussion that revolved around why some people fail to engage in the health‐promoting offer of group‐based self‐management support. The group participants agree that people with type 2 diabetes tend not to take their disease seriously until it has become severe. In this section of the interview, health professionals agree with each other and finish each other's sentences. The statement is presented while several are talking at once. Later, when the discussion has calmed down, the following statement is made:

I think that the setting has a lot to do with it, actually. Sometimes just a few words may actually change the setting, you know. We keep asking ourselves: have we been too harsh or too nice with the patient? (…) Sometimes they are just not receptive, and it is impossible to figure out what we have done wrong. We often ask ourselves the question why patients are not taking us seriously (…). (1)

In this statement, the act of conveying medical guidelines to the patient and the commensurate degree of “compliance” seem to govern the logic. The statements lean towards an understanding in which the health professional is viewed as the legitimate holder of valued information, which is supposed to be conveyed to the patient, who should then absorb the information uncritically.^34^ We may also see the above statement as imbued with a paternalistic approach, such that the patient seems to be under the guardianship of the professional while the professional wonders (similarly to a parent) if he/she is “too harsh or too nice.”

The priority of expert‐based knowledge, in line with a medical perspective and, hence, a medical logic, is also visible in the way group participants evaluate health‐promoting measures:

What is supposed to be quality and quality assurance, you get that in the health service, while in the voluntary group‐based support, it may be more random if you get a quality‐assured measure or not. I know that the Norwegian Diabetes Association is schooling their group leaders, and that, of course, is a way to ensure quality, but this will certainly vary between different group‐based support activities. (7)

The statement is situated in a context where health professionals express a concern that health‐promoting measures driven by voluntary organizations may not be “good” enough as an arena for ensuring lifestyle change. The political incentive to offer group‐based self‐management support, understood by health professionals in the focus groups as health education, is seen as a challenge in terms of limited time and space to support people in the demanding dietary regimens and lifestyle changes they are supposed to make. Health professionals expressed worry that health‐promoting initiatives that do not involve guidelines associated with expert knowledge may not facilitate health improvement. Even though the health professionals in our study express a high degree of critical reflection regarding the medical perspective, the quotation above indicates that the dominant logic or rationale is still occupied by the need for quality assurance and assurance that medical guidelines for compliance may be given to people with long‐term conditions.

When asking questions in the focus groups, we mentioned measures driven by private nonprofit organizations, such as the Norwegian Diabetes Association, in order to give the participants a broad perspective on health promotion. In line with earlier research, the health professionals in our study struggle with grasping that health promotion may encapsulate other frameworks for good living than that of medico‐centred health education.^32^ Even though volunteer organizations are mentioned in passing, the discussions in our focus groups are still centred on disease prevention. Organizations that have existed for decades in the Norwegian community that are important for well‐being and health in the population, such as the Norwegian Confederation of Sport or the Norwegian Trekking Association, are not discussed as health‐promoting arenas facilitating support. The health professionals in our study thereby show how they remain rooted in a medical perspective, striving to widen their approach to involve, for example, other community organizations.

### 3.3 Individual‐ or group‐oriented lifestyle change

In the following section, we try to illustrate how the logic of fellowship takes priority over the medical perspective approach, as health professionals imply that people with type 2 diabetes may help one another within the sphere of a group‐based health‐promoting setting. At the same time, while pointing out the benefit of the group‐based approach, health professionals appear to focus discussion of the group‐based measure on individual lifestyle choices.

Well, then, they have something in common, right? It is in a way the same thing: when I think about it, if they struggle with obesity, then they have at least that in common. Then, it is not a composition of different diseases. What is important especially with regard to patients with type 2 diabetes is that they are actually a stigmatized group. It may not be that easy to get support from others because the disease is like this: ‘You may have caused it yourself.’ So I think it is even more important for this patient group to meet others who understand what it is like. (4)

The statement suggests that the common features of the patient group are perceived to be obesity and stigma and that this commonality carries the potential to elicit support among the participants. However, the statement lacks a component of reflexivity, such that the composition is seen as categorizing a group of people based on negative features.^53^

The statement may also suggest that the health‐promoting setting is a supportive arena where concepts of stigma are addressed and where recognition and confirmation from others who have experienced the same challenges are important. Statements emphasizing the importance of connectivity and fellowship per se may be interpreted as a shift from a more dyadic medical perspective. However, the discussion also expresses a criticism of the group orientation:

“(…) You know, the groups may become very exclusive. Once they get established, then it may be difficult to join as a new member.” (7) (The statement is not challenged by any of the other focus group participants but is followed by a similar comment.)

The context of this statement is a discussion among health professionals considering whether the health‐promoting measures in the community actually make a difference in people's health, irrespective of the fact that they seem important for those who attend group‐based self‐management support. While discussing whether health‐promoting measures are good enough, one of the participants stated the following:

In order to participate in a [health‐promoting] offer, you should acknowledge that you actually have been given a diagnosis. It means that you have to take that into consideration (…). (3)

The above statement reflects, once again, the strength of the medical perspective, here illustrated as a focus on the disease, stating that in order to benefit from group‐based self‐management support, people with type 2 diabetes should base their participation on having a diagnosis.

There are seemingly positive statements regarding the group composition, as long as it will enhance individual lifestyle changes:

The exercise groups, I believe, may have the potential to help participants motivate each other. Members may feel that they can't let the group down. As long as you are more than just one individual, then it gives you motivation to actually perform better than if you were alone. It is a positive push. (4)

This statement highlights the potential of group‐based support to compel patients to participate in healthy activities via feelings of commitment to the other group members. Nevertheless, the point of “healthy activities” (exemplified here by physical exercise) becomes apparent, along with the “push” to “perform better at being healthy.” The moral imperative of performing better and the benefit of a guiltless conscience lead to the individual, person‐oriented lifestyle change that is sought to be accomplished by the group method. We thus understand that it is not the group setting per se that seems important, but rather the potential of the group to drive individual lifestyle change.

## 4 DISCUSSION

The health professionals’ discussions in the focus groups of our study suggest a high degree of self‐reflection when the value of health‐promoting measures was discussed. We use the term “self‐reflection” to describe those instances where group participants critically assess practices that we see as based on a medical perspective. Overall, the discussions in the focus groups revealed a picture of logics based on values that seem to be in opposition. There are instances where focus group participants seemed “stuck” in a medical perspective, even though they talk about changing frames. The medical perspective constituted an underlying frame of reference and an underlying shared logic in the focus groups. We have shown how health professionals tend to move and negotiate between prevention based on the logic of the medical perspective on the one hand and health promotion on the other hand. The concepts of disease prevention and health promotion are frequently described as being poorly differentiated^27^ in the literature. Particularly with regard to self‐management support measures, both health promotion and illness prevention are legitimatized through the objective of reducing the risk of disease or the worsening of already‐existing illnesses.^54^

Previous literature concerned with type 2 diabetes patients and health professionals highlights the distinction of “illness vs life,” where the main conflict between health professionals and diabetes patients is termed “keeping life and disease apart.”^55^, ^56^ In our study, we find that it is lifestyle vs life that has become the apparent category; that is, the notion of “life” is reduced to “lifestyle” based on health professionals’ understanding. The health professionals in our study seem mainly occupied with what they believe “works” for the assessment of a healthier lifestyle, not with what the individuals (patients) themselves see as a better life. This finding is in line with earlier research showing that professionals do not acknowledge the social and political conditions in which health‐related experiences unfold but rather adopt an individualistic approach and present self‐management as a question of control and of “bossing one's own mind.”^57^

Earlier research has shown that facilitators of group‐based self‐management support often do not explicitly focus on lifestyle change and instead wish to provide group participants with support and help them to achieve a high quality of life.^16^, ^58^ In contrast, it is possible to understand the findings in our study in line with research showing that health professionals revert to define health on a general level in terms of improvement and repair.^59^, ^60^, ^61^ Health‐promoting support that is not based on medical goals may bring substantial improvements for people with type 2 diabetes, while they simultaneously fail to achieve good health according to the medical definition.^59^

Nevertheless, it seems that health professionals express a lifestyle‐driven logic that aspires to incorporate a health‐promoting logic. This new framing, as mentioned by health professionals in addition to the focus group setting, may have given the health professionals in our study the freedom to engage in critical reflection of the existing systems of practice while simultaneously maintaining the logic of a medical perspective.

## 5 LIMITATIONS, STRENGTHS AND IMPLICATIONS FOR FURTHER RESEARCH

Our study participants represent unique cases, as there are not yet many health professionals who work in group‐based self‐management support measures in the community setting in Norway (at the time of data analysis, 2016). We recruited a variety of professionals who represent a wide range of health fields. Knowledge on how health professionals perceive health promotion and group‐based self‐management support is important for the adjustment of actors in the health‐promoting field in a way that benefits patients.

Hughes et al^16^ address the importance of the coproduction of meaning. Through the review on facilitators’ and participants’ experiences of group‐based self‐management support, the authors highlight that by focusing on medical aspects of self‐management, the groups constrain opportunities to provide support, and facilitators appear to lack the confidence necessary to support participants beyond a medical paradigm. By exploring further the notion of the coproduction of meaning beyond the professional‐patient interaction, Hughes et al direct attention to the broader set of ties with others, represented by each of the patient's relationships that may contribute to self‐management support. However, in our study, this approach may be relevant for directing attention to the importance of a social constructivist perspective in the health‐promoting field.

The findings in our study are based on few informants, and any generalizations should be made with caution. However, health professionals represent a network of other health professionals, and it is reasonable to believe that the dominance of a medical perspective in the health‐promoting setting is worth addressing for the benefit of people who take part in group‐based self‐management support.

## CONFLICT OF INTEREST

The authors declare no conflict of interest regarding this research.

## AUTHORS’ CONTRIBUTIONS

The first author has made substantial contribution to conception and design and also contributed to the acquisition of data. All authors have made substantial contribution to analysis and interpretation of data. While all authors have been involved in drafting the manuscript or revising it critically for important intellectual content, the first author has been responsible for appropriate incorporation of changes. All authors have given final approval of the version to be published. All authors have participated sufficiently in the work to take public responsibility for appropriate portions of the content. All authors have agreed to be accountable for all aspects of the work ensuring that questions related to the accuracy and integrity of any part of the work are appropriately investigated and resolved.

## References

[1] Lorig KR , Holman H . Self‐management education: history, definition, outcomes, and mechanisms. Ann Behav Med. 2003;26:1‐7. 12867348 10.1207/S15324796ABM2601_01

[2] Bodenheimer T , Lorig K , Holman H , Grumbach K . Patient self‐management of chronic disease in primary care. JAMA. 2002;288:2469‐2475. 12435261 10.1001/jama.288.19.2469

[3] Knutsen IR , Foss C . Caught between conduct and free choice‐a field study of an empowering programme in lifestyle change for obese patients. Scand J Caring Sci. 2011;25:126‐133. 20518867 10.1111/j.1471-6712.2010.00801.x

[4] Wild SH , Roglic G , Green A , Sicree R , King H . Global prevalence of diabetes: estimates for the year 2000 and projections for 2030: response to Rathman and Giani. Diabetes Care. 2004;27:2569. 15111519 10.2337/diacare.27.5.1047

[5] Kendall E , Ehrlich C , Sunderland N , Muenchberger H , Rushton C . Self‐managing versus self‐management: reinvigorating the socio‐political dimensions of self‐management. Chronic Illn. 2011;7:87‐98. 20921037 10.1177/1742395310380281

[6] Beaglehole R , Bonita R . Global Public Health: A New Era. Oxford, UK: Oxford University Press; 2009.

[7] Masseria C , Irwin R , Thomson S , Gemmill M , Mossialos E . Primary care in Europe. The London School of Economics and Political Science, Policy Brief 2009, European Commission, Directorate‐General Employment, Social Affairs and Equal Opportunities, Unit E1 – Social and Demographic Analysis

[8] Ham C . The ten characteristics of the high‐performing chronic care system. Health Econ Policy Law. 2010;5:71‐90. 19732475 10.1017/S1744133109990120

[9] Greene J , Hibbard JH , Sacks R , Overton V , Parrotta CD . When patient activation levels change, health outcomes and costs change, too. Health Aff. 2015;34:431‐437. 10.1377/hlthaff.2014.0452 25732493

[10] Agardh E , Allebeck P , Hallqvist J , Moradi T , Sidorchuk A . Type 2 diabetes incidence and socio‐economic position: a systematic review and meta‐analysis. Int J Epidemiol. 2011;40:804‐818. 21335614 10.1093/ije/dyr029

[11] Connolly V , Unwin N , Sherriff P , Bilous R , Kelly W . Diabetes prevalence and socioeconomic status: a population based study showing increased prevalence of type 2 diabetes mellitus in deprived areas. J Epidemiol Community Health. 2000;54:173‐177. 10746110 10.1136/jech.54.3.173 PMC1731634

[12] Albert C , Davia MA . Education is a key determinant of health in Europe: a comparative analysis of 11 countries. Health Promot Int. 2010;26:163‐170. 20935091 10.1093/heapro/daq059

[13] Kennedy A , Rogers A , Vassilev I , et al. Dynamics and nature of support in the personal networks of people with type 2 diabetes living in Europe: qualitative analysis of network properties. Health Expect. 2015;18:3172‐3185. 25393694 10.1111/hex.12306 PMC5810651

[14] Koetsenruijter J , van Eikelenboom N , van Lieshout J , et al. Social support and self‐management capabilities in diabetes patients: an international observational study. Patient Educ Couns. 2016;99:638‐643. 26549171 10.1016/j.pec.2015.10.029

[15] Koetsenruijter J , van Lieshout J , Lionis C , et al. Social support and health in diabetes patients: an observational study in six European countries in an era of austerity. PLoS One. 2015;10:e0135079. 26305559 10.1371/journal.pone.0135079 PMC4549295

[16] Hughes S , Lewis S , Willis K , Rogers A , Wyke S , Smith L . The experience of facilitators and participants of long term condition self‐management group programmes: a qualitative synthesis. Patient Educ Couns. 2017;100:2244‐2254. 28711415 10.1016/j.pec.2017.06.035

[17] Odgers‐Jewell K , Hughes R , Isenring E , Desbrow B , Leveritt M . Group facilitators’ perceptions of the attributes that contribute to the effectiveness of group‐based chronic disease self‐management education programs. Nutr Diet. 2015;72:347‐355.

[18] van der Does AM , Mash R . Evaluation of the “Take Five School”: an education programme for people with type 2 diabetes in the Western Cape, South Africa. Prim Care Diabetes. 2013;7:289‐295. 23932381 10.1016/j.pcd.2013.07.002

[19] Kousoulis AA , Patelarou E , Shea S , et al. Diabetes self‐management arrangements in Europe: a realist review to facilitate a project implemented in six countries. BMC Health Serv Res. 2014;14:453. 25278037 10.1186/1472-6963-14-453 PMC4283086

[20] Berenguera A , Pons‐Vigues M , Moreno‐Peral P , et al. Beyond the consultation room: proposals to approach health promotion in primary care according to health‐care users, key community informants and primary care centre workers. Health Expect. 2017;20:896‐910. 28116774 10.1111/hex.12530 PMC5600227

[21] Pelicand J , Fournier C , Le Rhun A , Aujoulat I . Self‐care support in paediatric patients with type 1 diabetes: bridging the gap between patient education and health promotion? A review. Health Expect. 2015;18:303‐311. 23311712 10.1111/hex.12041 PMC5060779

[22] Rafael AR . From rhetoric to reality: the changing face of public health nursing in southern Ontario. Public Health Nurs. 1999;16:50‐59. 10074822 10.1046/j.1525-1446.1999.00050.x

[23] Martinussen PE , Magnussen J . Resisting market‐inspired reform in healthcare: the role of professional subcultures in medicine. Soc Sci Med. 2011;73:193‐200. 21689875 10.1016/j.socscimed.2011.04.025

[24] Reay T , Hinings CR . Managing the rivalry of competing institutional logics. Organ Stud. 2009;30:629‐652.

[25] Ashcroft R . Health promotion and primary health care: examining the discourse. Soc Work Public Health. 2015;30:107‐116. 25375065 10.1080/19371918.2014.938395

[26] Nutbeam D . Evaluating health promotion—progress, problems and solutions. Health Promot Int. 1998;13:27‐44.

[27] Breslow L . From disease prevention to health promotion. JAMA. 1999;281:1030‐1033. 10086439 10.1001/jama.281.11.1030

[28] Herbert C , Visser A , Green L . Clinical health promotion and family physicians. Patient Educ Couns. 1995;25:223‐226. 7630825 10.1016/0738-3991(95)00812-e

[29] Knutsen IR , Terragni L , Foss C . Morbidly obese patients and lifestyle change: constructing ethical selves. Nurs Inq. 2011;18:348‐358. 22050620 10.1111/j.1440-1800.2011.00538.x

[30] Bossy D , Knutsen IR , Rogers A , Foss C . Group affiliation in self‐management: support or threat to identity? Health Expect. 2017;20:159‐170. 26868829 10.1111/hex.12448 PMC5217888

[31] Portillo MC , Regaira E , Pumar‐Méndez MJ , et al. Voluntary organizations and community groups as new partners in diabetes self‐management and education: a critical interpretative synthesis. Diabetes Educ. 2015;41:550‐568. 26160829 10.1177/0145721715594026

[32] Whitehead D . Health promotion in nursing: a Derridean discourse analysis. Health Promot Int. 2011;26:117‐127. 21131628 10.1093/heapro/daq073

[33] Jackson L . Health and health promotion In: Wills J, ed. Promoting Health (Vital Notes for Nurses). Oxford, UK: Blackwell Publishing; 2011:11‐28.

[34] Skelton AM . Patient education for the millennium: beyond control and emancipation? Patient Educ Couns. 1997;31:151‐158. 9216356 10.1016/s0738-3991(96)00986-x

[35] Casey D . Findings from non‐participant observational data concerning health promoting nursing practice in the acute hospital setting focusing on generalist nurses. J Clin Nurs. 2007;16:580‐592. 17335534 10.1111/j.1365-2702.2006.01557.x

[36] Irvine F . Examining the correspondence of theoretical and real interpretations of health promotion. J Clin Nurs. 2007;16:593‐602. 17335535 10.1111/j.1365-2702.2005.01539.x

[37] Kemppainen V , Tossavainen K , Turunen H . Nurses’ roles in health promotion practice: an integrative review. Health Promot Int. 2013;28:490‐501. 22888155 10.1093/heapro/das034

[38] Caraher M . Patient education and health promotion: clinical health promotion—the conceptual link. Patient Educ Couns. 1998;33:49‐58. 9481348 10.1016/s0738-3991(97)00055-4

[39] Dunn MB , Jones C . Institutional logics and institutional pluralism: the contestation of care and science logics in medical education, 1967–2005. Adm Sci Q. 2010;55:114‐149.

[40] Thornton PH , Ocasio W , Lounsbury M . The Institutional Logics Perspective: A New Approach to Culture, Structure, and Process. Oxford, UK: Oxford University Press; 2012.

[41] Scheller‐Kreinsen D , Blümel M , Busse R . Chronic disease management in Europe. Eurohealth (Lond). 2009;15:1.

[42] Wilkinson S . Focus Groups in Feminist Research: Power, Interaction, and the Co‐Construction of Meaning. London, UK: Women's Studies International Forum, Elsevier; 1998:111‐125.

[43] Haldar M , Engebretsen E , Album D . Legitimating the illegitimate: how doctors manage their knowledge of the prestige of diseases. Health (London). 2016;20:559‐577. 26245482 10.1177/1363459315596798

[44] Kitzinger J . Focus groups In: Carr A, Hewlett S, Hughes R, et al., eds. Qualitative Research in Health Care, 3rd edn. Oxford, UK: Wiley‐Blackwell; 2007:21‐31.

[45] Barbour RS . Making sense of focus groups. Med Educ. 2005;39:742‐750. 15960795 10.1111/j.1365-2929.2005.02200.x

[46] Fern EF . The use of focus groups for idea generation: the effects of group size, acquaintanceship, and moderator on response quantity and quality. J Mark Res. 1982;19:1‐13.

[47] Carlsen B , Glenton C . What about N? A methodological study of sample‐size reporting in focus group studies. BMC Med Res Methodol. 2011;11:26. 21396104 10.1186/1471-2288-11-26 PMC3061958

[48] Lehoux P , Poland B , Daudelin G . Focus group research and “the patient's view”. Soc Sci Med. 2006;63:2091‐2104. 16797811 10.1016/j.socscimed.2006.05.016

[49] Seale C , Gobo G , Gubrium JF , Silverman D . Qualitative Research Practice. London, UK: Sage; 2004.

[50] Braun V , Clarke V . Using thematic analysis in psychology. Qual Res Psychol. 2006;3:77‐101.

[51] Halkier B . Focus groups as social enactments: integrating interaction and content in the analysis of focus group data. Qual Res. 2010;10:71‐89.

[52] Bossy D , Knutsen IR , Rogers A , Foss C . Institutional logic in self‐management support: coexistence and diversity. Health Soc Care Community. 2016;24:e191‐e200. 26429669 10.1111/hsc.12277

[53] Robertson A . Shifting discourses on health in Canada: from health promotion to population health. Health Promot Int. 1998;13:155‐166.

[54] Fullagar S . Governing the healthy body: discourses of leisure and lifestyle within Australian health policy. Health (London). 2002;6:69‐84.

[55] Zoffmann V , Kirkevold M . Life versus disease in difficult diabetes care: conflicting perspectives disempower patients and professionals in problem solving. Qual Health Res. 2005;15:750‐765. 15961873 10.1177/1049732304273888

[56] Foss C , Knutsen I , Kennedy A , et al. Connectivity, contest and the ties of self‐management support for type 2 diabetes: a meta‐synthesis of qualitative literature. Health Soc Care Community. 2016;24:672‐686. 26429546 10.1111/hsc.12272

[57] Gomersall T , Madill A , Summers LK . A metasynthesis of the self‐management of type 2 diabetes. Qual Health Res. 2011;21:853‐871. 21429946 10.1177/1049732311402096

[58] Costello JF . Roles and strategies of diabetes support group facilitators: an exploratory study. Diabetes Educ. 2013;39:178‐186. 23411655 10.1177/0145721713476347

[59] Mol A . Proving or improving: on health care research as a form of self‐reflection. Qual Health Res. 2006;16:405‐414. 16449689 10.1177/1049732305285856

[60] Franklin M , Lewis S , Willis K , Rogers A , Venville A , Smith L . Controlled, constrained, or flexible? How self‐management goals are shaped by patient–provider interactions. [published online ahead of print June 26, 2018] Qual Health Res. 10.1177/1049732318774324 29871583

[61] Franklin M , Lewis S , Willis K , Bourke‐Taylor H , Smith L . Patients’ and healthcare professionals’ perceptions of self‐management support interactions: systematic review and qualitative synthesis. Chronic Illn. 2017;14:79‐103. 28530114 10.1177/1742395317710082

